# Multiplexed MRM-Based Proteomics Identified Multiple Biomarkers of Disease Severity in Human Heart Failure

**DOI:** 10.3390/ijms22020838

**Published:** 2021-01-15

**Authors:** Maura Brioschi, Erica Gianazza, Piergiuseppe Agostoni, Beatrice Zoanni, Alice Mallia, Cristina Banfi

**Affiliations:** 1 Centro Cardiologico Monzino, IRCCS, 20138 Milano, Italy; maura.brioschi@ccfm.it (M.B.); erica.gianazza@ccfm.it (E.G.); piergiuseppe.agostoni@ccfm.it (P.A.); beatrice.zoanni@ccfm.it (B.Z.); alice.mallia@ccfm.it (A.M.); 2 Dipartimento di Scienze Cliniche e di Comunità, Sezione Cardiovascolare, Università di Milano, 20122 Milano, Italy

**Keywords:** heart failure, proteomics, mass spectrometry, Multiple Reaction Monitoring (MRM)

## Abstract

Heart failure (HF) is a complex disease due to the intricate interplay of several mechanisms, which therefore implies the need for a multimarker strategy to better personalize the care of patients with HF. In this study, we developed a targeted mass spectrometry approach based on multiple reaction monitoring (MRM) to measure multiple circulating protein biomarkers, involved in cardiovascular disease, to address their relevance in the human HF, intending to assess the feasibility of the workflow in the disease monitoring and risk stratification. In this study, we analyzed a total of 60 plasma proteins in 30 plasma samples from eight control subjects and 22 age- and gender- matched HF patients. We identified a panel of four plasma proteins, namely Neuropilin-2, Beta 2 microglobulin, alpha-1-antichymotrypsin, and complement component C9, that were more abundant in HF patients in relation to disease severity and pulmonary dysfunction. Moreover, we showed the ability of the combination of these candidate proteins to discriminate, with sufficient accuracy, HF patients from healthy subjects. In conclusion, we demonstrated the feasibility and potential of a proteomic workflow based on MRM mass spectrometry for the evaluation of multiple proteins in human plasma and the identification of a panel of biomarkers of HF severity.

## 1. Introduction

Heart failure (HF) is a complex disease in which the intricate interplay of several mechanisms, including neurohormonal activation, inflammation, myocardial stretch, matrix remodeling, and myocyte injury, contributes to its development and progression [1]. Additionally, the physiopathological interactions between the heart and other organs, such as the kidneys, is one of the most important factors contributing to the disease complexity. Further, HF diagnosis is mainly based on clinical signs and symptoms, with the natriuretic peptides, brain natriuretic peptide (BNP) and NT-proBNP, being the most commonly used biomarkers for the diagnosis and prognostication of patients with HF. Unfortunately, neither peptide is a perfect surrogate for the presence or severity of HF, and their limitations are well discussed in a recent review by Ibrahim et al. [2]. 

For the above-mentioned reasons, the search for biomarkers for the management of patients with HF has increased tremendously over the past few years, and many new biomarkers are currently under investigation. At present, they appear to have a synergistic role along with the natriuretic peptides, both in terms of diagnosis and determination of prognosis. Multimarker strategies have indeed the potential to better personalize the care of patients with HF. Further, a multimarker approach incorporates the simultaneous assessment of several biomarkers to identify the activity of multiple different pathophysiological pathways and thereby provide integrated information concerning the state of the patient. There is evidence that this combined strategy leads to improved measurement of HF risk compared to traditional risk scores [3,4,5,6]. A multi-biomarker approach is also suggested to identify high-risk patients for stratification and prevention [7,8].

In this context, recent advances in the field of proteomics, based on mass spectrometry (MS), have provided novel approaches for the simultaneous quantitation of multiple protein biomarkers. The selected reaction monitoring (SRM)/multiple reaction monitoring (MRM) approach used in tandem MS on a triple quadrupole mass spectrometer applied to proteins allows a fine detection of peptides, derived from a protein of interest, with a high level of specificity and sensitivity [9]. Indeed, MRM reproducibly measures the concentration of multiple analytes when stable isotope-labeled internal standards are included in the workflow as demonstrated in decades of application of this methodology in the field of low molecular weight molecules, such as drugs or environmental contaminations [9].

In this study, we employed a targeted MRM approach to measure multiple circulating proteins involved in cardiovascular disease to address their relevance in the human HF, intending to assess the feasibility of the workflow in the disease monitoring and risk stratification.

## 2. Results

### 2.1. Characteristics of Study Participants

In this study, we analyzed 30 plasma samples from 8 control subjects (52.1 ± 3.3 years old) and 22 age- and gender- matched HF patients (59 ± 10.27 years old, *p* = 0.057 vs. controls) with reduced left ventricular ejection fraction (LVEF), in stable clinical conditions, following standard therapy. HF etiology was cardiomyopathy due to ischemic coronary disease in 11 cases and not-ischemic in the other cases. Among HF subjects, 10 were in New York Heart association (NYHA) class I–II and 12 in class III–IV. The mean LVEF in HF patients was 30.7 ± 5.8%; the median BNP was 304 pg/mL (lower quartile: 108 pg/mL; upper quartile: 482 pg/mL). All patients underwent a cardiopulmonary exercise test and spirometry to obtain a clinical characterization of the severity of HF, based on pulmonary function. In particular, the levels of VO_2_/Kg, an index of oxygen consumption that reflects HF severity, were used to stratify HF patients [7] as reported in Table S1, which summarizes the clinical and demographical characteristics of the study population.

### 2.2. Analysis of Plasma Biomarkers in Relation to HF Severity

**Figure 1:**
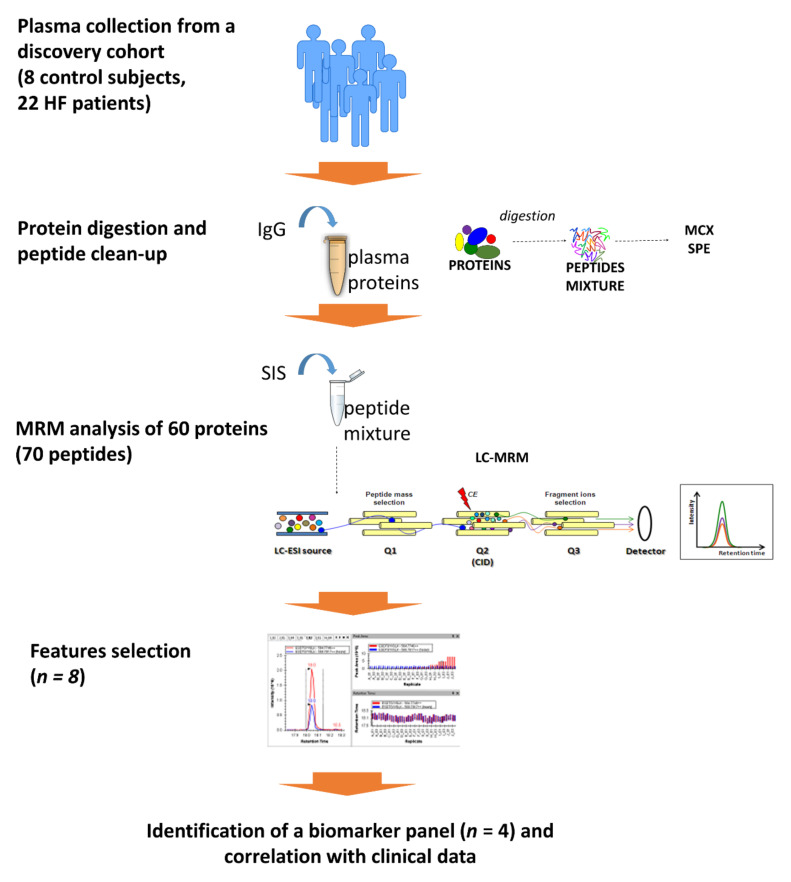
Workflow of the study.

Here, we developed a multiplexed targeted liquid chromatography mass spectrometry (LC-MS)-based assay to quantitatively measure multiple plasma proteins with potential roles in cardiovascular diseases, and we applied it to a cohort of HF patients to identify potential biomarkers of HF severity. The workflow of this study is summarized in Figure 1.

Plasma proteins were directly digested with trypsin without any immunodepletion step and subsequently purified by MCX solid phase extraction (SPE), to eliminate interfering molecules. A known amount of an immunoglobulin (IgG) was added to each sample at the beginning of the workflow and was analyzed by MRM with a dedicated method, as described in the method section, to evaluate the reproducibility of the entire protocol. A coefficient of variation of 11.24%, 12.5%, and 13.04% was calculated for the three quantified transitions of the IgG among all the analyzed plasma samples.

The development of an MRM method for the detection of the 76 protein candidates was performed with stable isotope labeled internal standards (SIS) added to a pool of plasma samples, and we, finally, set up a scheduled method that allowed us to evaluate a total of 70 peptides corresponding to 60 proteins (Table S2). We considered for the quantitative analysis only those peptides with at least two coeluting transitions per peptide, excluding those peptides that were undetectable, detected by a single transition, or without a linear response in the standard curve.

**Figure 2:**
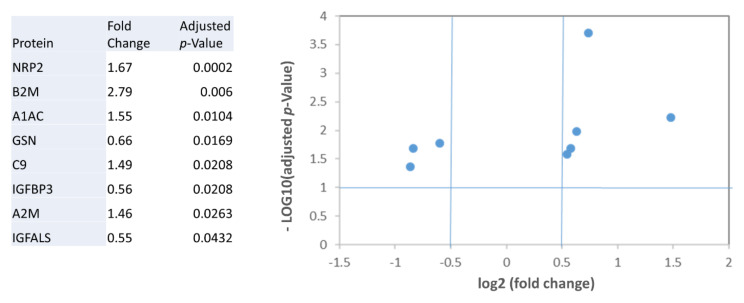
Differentially expressed proteins in patients with severe HF (VO_2_/Kg < 12 mL/min/Kg) in relation to control subjects. Table and graphical representation in a Vulcano plot of the fold changes, and statistical significance for the proteins with different levels after adjustment for multiple testing.

Thus, we performed a preliminary features selection based on those proteins that were significantly different, both with univariable and multivariable analysis, between control subjects and those patients with the highest level of HF severity, that is patients with levels of VO_2_/Kg < 12 mL/min/Kg. As reported in Figure 2, according to this analysis, we selected Neuropilin-2 (NRP2), Beta-2-microglobulin (B2M), Alpha-1-antichymotrypsin (A1AC), Gelsolin (GSN), Complement component C9 (C9), Insulin-like growth factor-binding protein 3 (IGFBP3), Alpha-2-macroglobulin (A2M), and Insulin-like growth factor-binding protein complex acid labile subunit (IGFALS). The above proteins were all significantly different between control subjects and severe HF patients, with a fold increase higher than 1.4. Considering all patients as a single group none of the analyzed proteins resulted to be significantly different from control subjects, due to the differences in terms of HF severity (data not shown).

To assess if these proteins were differentially expressed in patients with different HF severity, we performed an ANOVA, including low severity HF patients (VO_2_/Kg > 16 mL/min/Kg), medium severity HF patients (12 < VO_2_/Kg <16 mL/min/Kg), and high severity HF patients (VO_2_/Kg < 12 mL/min/Kg). We identified NRP2, B2M, A1AC, GSN, C9, as significantly different among groups (Table 1). These data were also confirmed with a general linear model, adjusting for age, due to the differences among groups for this variable (Tables S1 and S3).

**Figure 3:**
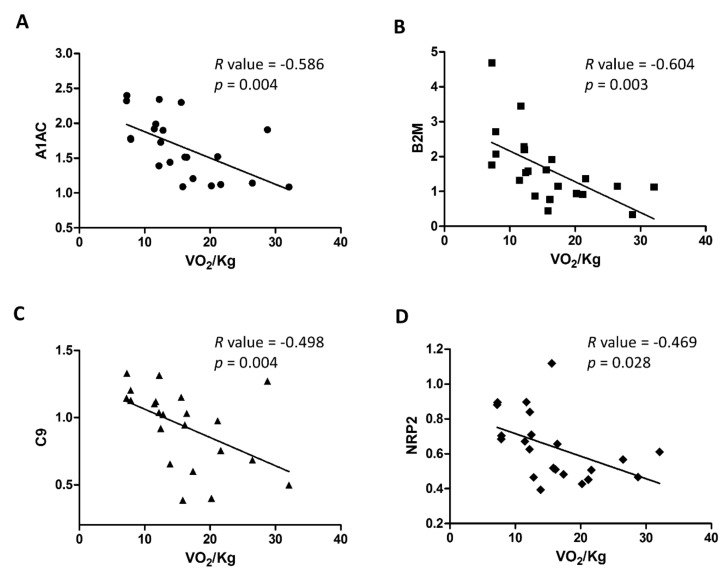
Correlation of selected features (**A**–**D**) with VO_2_/Kg.

To better clarify the link between the selected features and HF severity, we evaluated the linear correlation of the 5 selected features with the levels of VO_2_/Kg, and demonstrated a significant inverse correlation for A1AC (*R*-value = −0.586, *p* = 0.004), B2M (*R*-value= −0.604, *p* = 0.003), C9 (*R*-value = −0.498, *p* = 0.018) and NRP2 (*R*-value = −0.469, *p* = 0.028) (Figure 3), while gelsolin did not result significantly correlated with the levels of VO_2_/Kg. Of note, only the correlation between B2M and the levels of VO_2_/Kg was independent from the levels of B-type natriuretic peptide (BNP).

### 2.3. Diagnostic Performance of the Plasma Biomarkers

**Figure 4:**
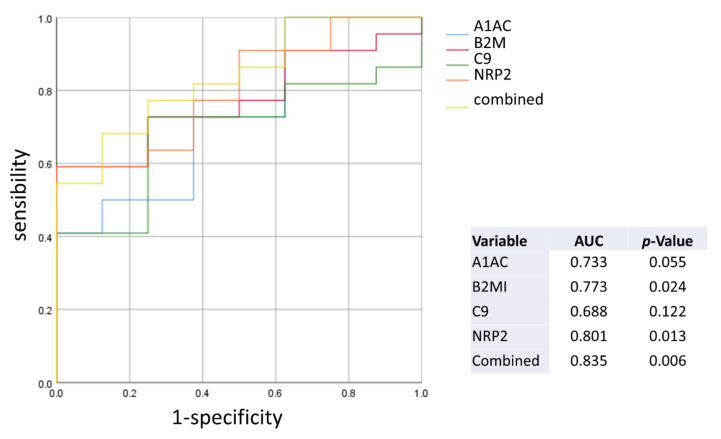
ROC generated with the selected features.

To test the diagnostic performance of the 4 selected features, which correlate with the severity of HF, we evaluated their discriminating capacity with a ROC analysis, both individually, and combined by linear regression, as shown in Figure 4. Of note, ROC, based on MRM data revealed that the combination of the four features can diagnose HF with a 68.2% sensitivity and 87.5% specificity (AUC 0.835, cut-off = 0.726), overcoming the performance of the biomarkers considered individually.

### 2.4. Interaction Network Analysis

**Figure 5:**
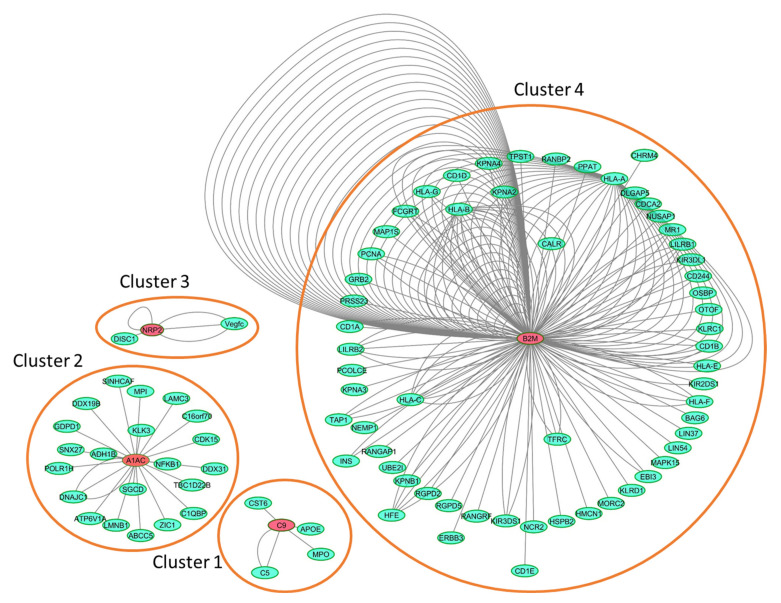
Human IntAct network generated with the four identified biomarkers and their interactors. Details on the network components are reported in Table S4.

To contextualize the four identified potential biomarkers, we created a human protein interaction network of the features and their interactors with Intact, including 220 binary interactions between 91 interactors (Figure 5). A gene ontology enrichment analysis performed with String highlighted the involvement of the majority of the interactors in the regulation of the immune system process (*p* = 4.57 × 10^−12^). More specifically, proteins belonging to cluster 4 are mainly involved in the regulation of the immune response (*p* = 1.38 × 10^−11^), while those from cluster 2 regulates signaling (*p* = 9.26 × 10^−5^) and cell communication (*p* = 9.26 × 10^−5^), while those from cluster 1 regulate the inflammatory response (*p* = 0.0043).

## 3. Discussion

In the present study, we applied a proteomic workflow based on MRM mass spectrometry for the evaluation of multiple protein biomarkers in human plasma, and we demonstrated its feasibility and potential for the identification of a panel of biomarkers of HF severity. Moreover, we showed the ability of the combination of these proteins to identify, with sufficient accuracy, HF patients from healthy subjects. To the best of our knowledge, this is the first study that applies a targeted proteomics analysis to study the levels of plasma proteins according to the severity of pulmonary dysfunction in HF patients with reduced ejection fraction. MRM is a mass spectrometry method that is emerging as the proteomic approach of choice for targeting selective peptides of a protein for their detection and highly specific quantification in biofluids. An advantage of this targeted workflow is that it does not imply the depletion of highly abundant proteins representing the majority of plasma proteins, an approach extensively used in discovery proteomic studies, but often associated with the risk of uncontrollable off-target depletion [10]. Further, compared with conventional ELISA, MRM assays have many advantages for the verification and validation of large numbers of biomarkers and the simultaneous direct measurement of panels of candidate biomarkers of disease, because it does not need the expensive, and time-consuming, development of specific antibodies. Thus, the MRM method for biomarker discovery plays a powerful biological role in the identification of candidate markers of pathophysiology and targets of drugs to treat diseases.

The approach developed in this study, allowed us to identify a panel of four plasma proteins, namely NRP2, B2M, A1AC, and C9, that are more abundant in HF patients correlating with pulmonary dysfunction and discriminating patients from healthy subjects.

Beyond the discriminating capacity of the identified protein panel, the creation of a human protein interaction network of the proteins and their interactors highlighted the involvement of the majority of the interactors in the regulation of the immune system process.

We found indeed an increase in the plasma levels of the protein complement C9. C9, which is the membrane attack complex (MAC)/perforin-like protein complement component 9, is the major component of the MAC, a multi-protein complex that forms pores in the membrane of target pathogens [11]. Our results confirm the finding from a previous study based on an aptamer-based platform, which included a detailed investigation of the protein components [12]. The authors found a marked increase of all effector proteins in subjects with HF. Additionally, they observed higher levels of complement proteins in the coronary sinus blood than in the peripheral blood, and a slight decline after transplantation, which overall suggest a cardiac complement activation in HF development [12].

Beta 2 microglobulin (B2M) is a part of the major histocompatibility complex, which is found on all nucleated human cells and thrombocytes. B2M was found to be elevated in high turnover states and infectious and chronic inflammatory diseases [13,14,15]. B2M was recently found to be independently and significantly associated with adverse cardiovascular outcomes in patients with prevalent asymptomatic carotid atherosclerosis [16]. It also predicted cardiovascular events better compared with high sensitivity- C reactive protein (hs-CRP) in older adults [17]. Further, it has been suggested that B2M could also accelerate and amplify, if not initiate, an inflammatory response, by stimulating infiltrating mononuclear cells, to secrete more proinflammatory cytokines [18]. Further, a proteomic study found plasma B2M to be a risk marker for coronary heart diseases in postmenopausal women [19]. Finally, in peripheral arterial disease patients, circulating B2M is elevated and correlates with the severity of disease independent of other risk factors.

Neuropilin-2 (NRP2), together with neuropilin-1 (NRP1), mainly act as co-receptors for class III Semaphorins and members of the vascular endothelial growth factor family. They are widely known for their role in a wide array of physiological processes, such as cardiovascular, angiogenesis, lymphangiogenesis, neuronal development, as well as various clinical disorders [20]. However, their interaction with several other ligands such as transforming growth factor β (TGF-β), Hepatocyte Growth Factor, and Platelet-derived growth factor, besides Semaphorin-3A and Vascular-Endothelial Growth Factor, has raised even more questions about their functions [20]. Further, NRP1 and NRP2 are also expressed in various immune cells, where they regulate the immune response, under normal physiological conditions and during pathological disorders. Of interest, in a recent study, neuropilin was predictive of rehospitalizations of HF [21].

Further, the role of the acute phase protein alpha-1-antichymotrypsin (A1AC), also known as SERPINA3 (serpin peptidase inhibitor, clade A, member 3), in HF has been scarcely investigated. A paper published in 2013 by Lok et al. [22] specifically addressed the role of A1AC in HF showing that its plasma levels were significantly elevated in end-stage HF patients and decreased to baseline after Left Ventricular Assist Devices support. Many hypotheses can be formulated on the potential role of A1AC in HF. A1AC might induce the proinflammatory cytokine tumor necrosis factor-α, and nuclear factor-κB [23], which are known to induce cardiomyocyte apoptosis as well as hypertrophic growth and cardiac remodeling [24]. Being the major inhibitor of the extracellular serine protease activity of Cathepsin G, A1AC is required for the maintenance of the connective tissue integrity of the heart. An imbalance in favor of Cathepsin G promotes indeed a pathological remodeling of the heart [25] through multiple mechanisms, among which the conversion of angiotensin I to angiotensin II (a pro-fibrotic and pro-inflammatory mediator) that ultimately activates the TGFβ-pathway, resulting in myocyte necrosis, hypertrophy, and increased fibrosis [26].

Considering the enormous societal burden of HF in terms of cost, morbidity, and mortality, there is an urgent need for laboratory testing to improve our understanding of the complex disease process of HF and possibly to personalize care through better individual phenotyping. The prevalence of acute and chronic HF is steadily increasing worldwide. Further, the disease presents significant differences between sexes, races, ages, and comorbidities, which dictate phenotypes, prognosis, and response to the disease treatment [27].

Circulating biomarkers certainly provide a low cost, low risk, and feasible method to confirm or exclude an HF diagnosis, help to establish prognosis in the diagnosis, and likely provide substantial information on the complex pathophysiology of the HF syndrome.

However, for complex diseases, such as HF, it is unrealistic that a single marker can reflect all of the features of this syndrome, whereas the combined use of more parameters would certainly give a more comprehensive insight into an individual patient [2]. To underline the complex nature of HF with the involvement of multiple organs, recent evidence indicates the beneficial effects of SGLT2 inhibition in patients with a broad spectrum of severity of HFrEF. Indeed, when added to all appropriate treatments for HF, SGLT2 inhibitors reduced all-cause and cardiovascular death, hospitalizations for HF, and serious adverse renal outcomes [28]. The underlying mechanisms that explain the beneficial effects of SGLT2 inhibition are multiple but may involve weight loss, reduction in adipose tissue inflammation, slight increase in ketone bodies, and diminution of uric acid levels or attenuation of oxidative stress [29]. Therefore, a multimarker panel that includes biomarkers representative of each pathophysiological pathway involved in the onset and progression of the disease, would represent the ideal solution [2]. However, this is challenging. First, the identification of truly novel biomarkers outside of known pathways is difficult and correlated biomarkers generally add little to risk prediction [30]. Indeed, in the past years, Wang et al. [31], measured 10 biomarkers in 3209 participants attending a routine examination cycle of the Framingham Heart Study (CRP, BNP, NT-proBNP, aldosterone, renin, fibrinogen, D-dimer, plasminogen-activator inhibitor type 1, homocysteine, and urinary albumin-to-creatinine ratio), and found that for assessing risk in individuals, the use of the 10 contemporary biomarkers only moderately added to standard risk factors. More recently, Chirinos et al. [32] measured 49 plasma biomarkers from TOPCAT (Treatment of Preserved Cardiac Function Heart Failure with an Aldosterone Antagonist) trial participants (*n* = 379) using a Multiplex assay to assess the relationship between biomarkers and the risk of all-cause death or HF-related hospital admission. In this case, the authors found that various novel circulating biomarkers in key pathophysiological domains are predictive of outcomes in HFpEF, and they concluded that a multimarker approach coupled with machine-learning represents a promising strategy for enhancing risk stratification in HFpEF.

Multimarker strategies have the potential to better personalize the care of patients with HF. It has been demonstrated by Gaggin et al. that a model containing traditional risk factors and several biomarkers including ET-1, NT-proBNP, hsTnI, and ST2 best-predicted cardiovascular events, and ET-1 improved classification in HF [33]. Therefore, the addition of other potential markers of HF to our panel is expected to aid in the diagnosis and risk stratification/prognosis in HF. To this list, we could add the plasma immature species of the lung-specific surfactant protein B (proSP-B), which represents a reliable marker of lung dysfunction in HF, correlates with prognosis, and represents a precocious marker of drug therapy [34,35,36,37,38]. Further, proSP-B binds to HDL, impairing their antioxidant capacity [39], thus likely contributing to the progression of the disease. Indeed, an impaired HDL antioxidative capacity is actually associated with higher mortality in HF patients independent of the traditional cardiovascular risk factors, irrespectively of the underlying etiology. Thus, additional research is warranted and is in progress to develop a multimarker model for HF. In light of the existing evidence, a multimarker approach is certainly suggested given the complex pathophysiological processes underlying HF.

Though our results were promising, we recognize several limitations. First, this was a single-center study, limited by the sample size, and restricted ethnicity. Thus, large-scale studies of individuals from various ethnic groups are needed to confirm our results. Second, we did not assess the impact of the HF etiology (i.e., ischemic and non-ischemic patients) or differences in the therapy or the presence of other comorbidities, and we did not explore HF patients with preserved ejection fraction in the current work, thus the detected biomarkers may not be unique to HF. Finally, the utility of candidate biomarkers was assessed at a single time point in stable HF versus healthy volunteers. Thus, the measurement of their levels, at different time periods, to assess their reproducibility in patients in stable conditions and the effect of treatments should be performed, as well as after a follow-up period to test also their prognostic value. Moreover, the low sample size and the lower number of healthy subjects might alter the estimation of the specificity resulting from the ROC curve. Another significant limitation of this manuscript is that it lacks the verification study of these four biomarkers based on the analysis of independent samples to verify the discovery study results. Therefore, we underline that the biomarker study presented in the manuscript is still in the preliminary stage.

Despite this limitation, the present study identified a panel of four protein biomarkers that correlate with HF severity and have a diagnostic power. Another advantage is represented by the use of plasma, a patient biofluid, that, compared with myocardial tissue, is less limited in sample access, obtained less invasively and easier to sample repeatedly and to process for storage and analysis in a more standardized and less complex manner.

Considering that patients with HF often present with signs and symptoms that are nonspecific and with a wide differential diagnosis, making the diagnosis by clinical presentation alone challenging. Indeed, some of the signs and symptoms (such as dyspnea, orthopnea, and paroxysmal nocturnal dyspnea), are heterogenous thus resulting in delays in definitive diagnosis and treatment, often linked with poor prognosis. The “traditional” clinical evaluation of patients with chronic HF involves a combination of medical history, physical examination, imaging techniques, bioelectrical impedance vector analysis, heart catheterization, which, in isolation, have a limited performance in HF patients care, because they are limited in sensitivity and specificity, have high costs and they are time consuming, and sometimes they are invasive (i.e., right heart catheterization) [40].

Thus, the introduction of objective, noninvasive, biologically meaningful biomarkers to clinical assessment has the potential to considerably change the way HF is diagnosed and monitored [2,8,41,42]. In conclusion, the development of this method makes potential further validation and signature refinement possible on other center cohorts in the future, as well as its application to disease models for novel therapy studies. As studies continue to emerge on the use of other novel biomarkers, we will undoubtedly witness their incorporation into future clinical practice guidelines as well. Given the complex physiology in HF, it is reasonable to expect that the future of biomarker testing lies in the application of multimarker testing panels, precision medicine to improve HF care delivery, and the use of biomarkers in proteomics and metabolomics to further improve HF care, accurately identifying patients at high risk and guiding therapies.

## 4. Materials and Methods

### 4.1. Study Population

This study was performed on plasma samples from a subset of healthy subjects (controls) and HF patients in stable clinical conditions, matched according to their age, sex, and clinical characteristics. All patients belong to a cohort of HF patients regularly followed at our HF Unit and underwent our standard HF assessment which included full clinical evaluation, standard laboratory tests, echocardiography, spirometry, and alveolar-capillary diffusion, as well as a cardiopulmonary exercise test, as previously described [34]. The study was approved by the Ethical Committee European Institute of Oncology and Monzino Cardiologic Center (registration number R391/16-CCM406, approved 19/02/2016) and all patients gave their informed consent before taking part in the study.

### 4.2. Sample Preparation

Plasma samples were digested using the ProteinWorks^TM^ eXpress Digest kit (Waters Corporation, Milford, MA, USA) according to the manufacturer’s instructions. Briefly, 35 µL of plasma were mixed with 12 µl of 200 µg/mL Intact mAb Mass Check Standard (Waters Corporation), a fully characterized monoclonal antibody used as an internal LC-MS standard for MS optimization and performance testing, and then diluted to a total volume of 120 µL with the digestion buffer provided by the kit. The sample was added to a cluster tube containing the dried denaturant and placed into the dry block heater at 80 °C for 10 min. Twenty microliters of reduction agent were then added to the sample and placed into the dry block heater at 60 °C for 20 min; followed by the addition of 30 µL of alkylation agent at room temperature in the dark for 30 min. At this stage, the sample was digested with 30 µL of trypsin solution and placed into the dry block heater at 45 °C for 2 h. Finally, 5 µL of the trypsin inactivation agent were added into the sample for an additional 15 min at 45 °C. The supernatant recovered by centrifugation of the sample at 3000 rpm for 15 min was stored at −80 °C for further clean-up by solid phase extraction (SPE). Protein digest clean-up was performed according to the ProteinWorks^TM^ µElution SPE Clean-up protocol (Waters Corporation, Milford, MA, USA). Briefly, the Oasis µElution MCX plate was placed on the vacuum manifold, conditioned with methanol and equilibrated with H_2_O before the loading of the digested sample. The plate was then washed with 2% formic acid in H_2_O and 5% methanol in H_2_O. Therefore, the elution of the sample was performed using a 60/40% H_2_O/acetonitrile (*v*:*v*) solution containing 2% ammonium hydroxide. The eluates were collected in a collection plate and diluted with the same volume of H_2_O to reduce the acetonitrile concentration thus maintaining the peak shape during the LC-MS analysis. Before the MS analysis, purified plasma samples were mixed with a mixture of stable isotope-labeled internal standards (SIS) for protein quantitation of a total of 76 proteins (101 peptides, Table S5), provided with the PeptiQuant™ Biomarker Assessment Kit (BAK-A6495-76-20, Cambridge Isotope Laboratories, Inc., Tewksbury, MA, USA).

### 4.3. Liquid Chromatography Mass Spectrometry (LC-MS) Analysis

Two microliters of each sample, containing 12.5 fmol/µL of SIS, were injected into a Xevo TQ-S micro triple quadrupole mass spectrometer coupled to a Waters ACQUITY ultra-performance liquid chromatography (UPLC) M-Class system through an ionKey source (Waters Corporation, Milford, MA, USA). The instrument was operated in positive ion mode in unit resolution. The capillary voltage was maintained at 3.80 kV with the source temperature held constant at 120 °C. After isocratic trapping for 1 min with a flow rate at 30 µL/min 99.5% solution A (99.9% LC-MS-grade water with 0.1% formic acid)/0.5% solution B (LC-MS-grade 99.9% acetonitrile with 0.1% formic acid) on a ACQUITY UPLC M-Class Symmetry C18 Trap Column, 100 Å, 5 µm, 300 µm × 50 mm (Waters Corporation, Milford, MA, USA) column, a iKey Peptide HSS T3 column, 100 Å 1.8 µm, 150 µm × 100 mm (Waters Corporation, Milford, MA, USA) was used for peptide separation. The flow rate was set to 3 µL/min with a column temperature of 60 °C. A gradient of solvent A and solvent B was applied with a total run time of 25 min as follows: 0–2.5 min at 2% B; 2.5–17.50 min linear increase from 2 to 90% B; 17.5–19.5 min 90% B; 20–25 min 2% B.

Transition selection and optimization of collision energy were performed using Skyline (v. 4.2). The peptides and transitions of the final biomarker panel are given in Table S2, while those for the analysis of control IgG are reported in Table S6. According to the retention time of the peptides, a scheduled method was set up to maintain the dwell time between 0.005 and 0.050 s. A pool of HF patients was analyzed for the quality controls throughout the run, and a coefficient of variation below 20% was considered acceptable. A standard curve 0.5–125 fmol/µL was run to test the linearity of the response for all the peptides using an increasing concentration of SIS in a pool of plasma protein digest. The accuracy of each standard point was calculated with TargetLynx based on the determined linear regression curve (1/x) and should be between 80% and 120% of the expected value. Low signal standard points are more likely to have accuracies outside this range and were sometimes excluded from the linear regression curve (Table S7). Moreover, the quality was further assessed by manually inspecting each peak in the data set with Skyline software and considering the relative dot product obtained comparing transition distribution in light and heavy peptides. Peptide quantity is expressed as the ratio of the integrated area of the endogenous peptide to the area of the corresponding SIS calculated with Skyline.

### 4.4. Computational Analysis

A protein interaction network was generated with IntAct (v. 4.2.15) [43] and visualized with Cytoscape (v. 3.5.1). Enriched biological processes for the component of the clusters were identified with the enrichment widget of STRING [44], reporting enrichment *p*-value after Hypergeometric test and Benjamini and Hochberg correction of multiple testing.

### 4.5. Statistical Analysis

Categorical variables are presented as frequencies and percentages, and continuous variables are mainly expressed as means ± SD. The data acquired by MRM analysis were analyzed using Skyline (v. 4.2) with manual inspection of peak integration, and the included MSstats tool was used for feature selection. Benjamini–Hochberg procedure has been used for multiple testing correction. All of the measured variables were analyzed separately for each group according to the severity of HF, assessed in terms of VO_2_/kg.

Statistical analyses were mainly performed using the SPSS software for Windows (IBM, Armonk, NY, USA, v. 25) and R 4.0.2. A Kolmogorov–Smirnov test was performed to test normal distribution of the variables, and non-normally distributed variables were log-transformed.

Significant differences between groups were evaluated by two-sided, unpaired Student’s *t*-test, or ANOVA. Pearson correlation was used to identify a possible correlation between selected features and clinical variables. A general linear model (GLM) was used to highlight the trend of increase of selected features with the severity of the disease, both as univariate analysis and as multivariate analysis taking into consideration differences of age. Finally, ROC analysis was performed with SPSS for single variables or multiple variables, combined with linear regression. A *p*-value lower than or equal to 0.05 was considered as statistically significant.

## Figures and Tables

**Table 1:** Levels of the selected features in healthy controls and HF patients, categorized according to different disease severity (VO_2_/Kg). *p*-values from ANOVA, and adjusted *p*-value obtained with a general linear model adjusting for age.

	Controls	Low Severity HF	Medium Severity HF	High Severity HF		
Protein		VO_2_/Kg > 16 mL/min/Kg (*n* = 9)	12 < VO_2_/Kg < 16 mL/min/Kg (*n* = 7)	VO_2_/Kg < 12 mL/min/Kg (*n* = 6)	*p*-Value	Adjusted *p*-Value
**A1AC**	1.332 ± 0.323	1.3466 ± 0.282	1.742 ± 0.471	2.030 ± 0.270	0.002	0.001
**A2M**	0.946 ± 0.163	1.055 ± 0.356	1.028 ± 0.169	1.398 ± 0.475	ns	-
**B2M**	0.873 ± 0.319	1.073 ± 0.429	1.5058 ± 0.663	2.665 ± 1.240	<0.001	0.001
**C9**	0.768 ± 0.192	0.7955 ± 0.282	0.9268 ± 0.314	1.171 ± 0.0854	0.020	0.014
**GSN**	1.034 ± 0.174	0.760 ± 0.185	0.822 ± 0.200	0.729 ± 0.216	0.020	0.043
**IGFBP3**	0.929 ± 0.0930	0.774 ± 0.124	0.849 ± 0.340	0.589 ± 0.335	ns	-
**IGFALS**	1.101 ± 0.251	0.851 ± 0.250	0.963 ± 0.437	0.696 ± 0.390	ns	-
**NRP2**	0.448 ± 0.0855	0.520 ± 0.076	0.667 ± 0.250	0.789 ± 0.112	0.001	< 0.001

## Data Availability

Data collected in the study will be made available using the data repository Zenodo (https://zenodo.org/) with restricted access upon request to Direzione.scientifica@ccfm.it.

## Supplementary Materials

The following are available online at https://www.mdpi.com/1422-0067/22/2/838/s1, Table S1: Clinical characteristics of the study population, Table S2: List of all the analyzed transitions for light and heavy peptides, Table S3: Clinical characteristics of each subject, Table S4: List of all the protein displayed in the IntAct generated network in Figure 5, Table S5: List of peptides present in the stable isotope-labeled standards mixture, Table S6: List of the analyzed transitions for the evaluation of IgG used as analytical control, Table S7. List of all the analyzed peptide with the results obtained from their standard curves and their relative dot product (light to heavy).
