# Supplementary figures and images for: Multiplexed MRM-Based Proteomics Identified Multiple Biomarkers of Disease Severity in Human Heart Failure
Source: Int J Mol Sci. 2021 Jan 15;22(2):838. doi: 10.3390/ijms22020838 (PMC7830442; doi:10.3390/ijms22020838)

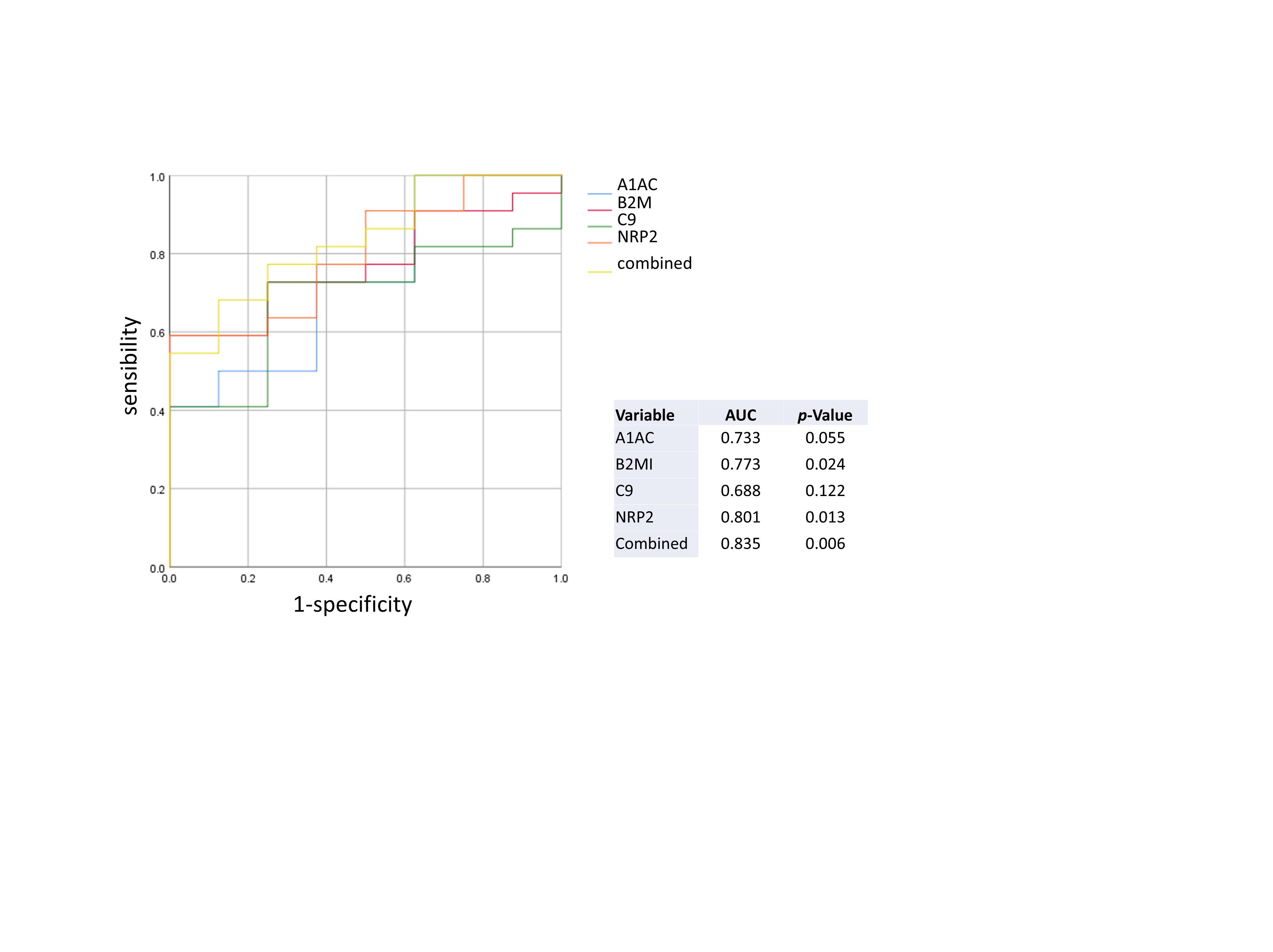

Supplement: Supplementary file 1 [file ijms-22-00838-s001.zip › Figure 4 $X.tif]

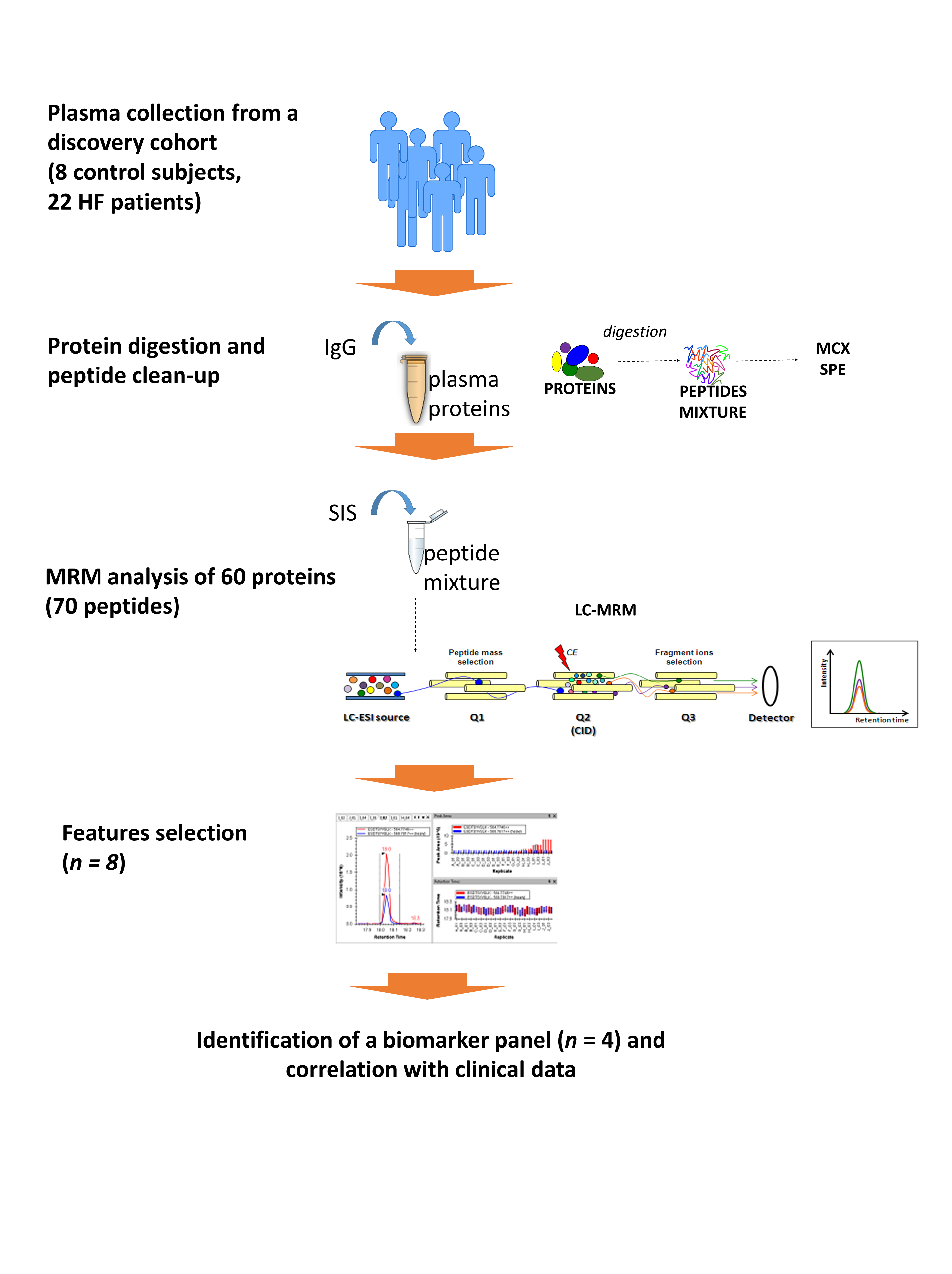

Supplement: Supplementary file 1 [file ijms-22-00838-s001.zip › Figure 1 4X.tif]

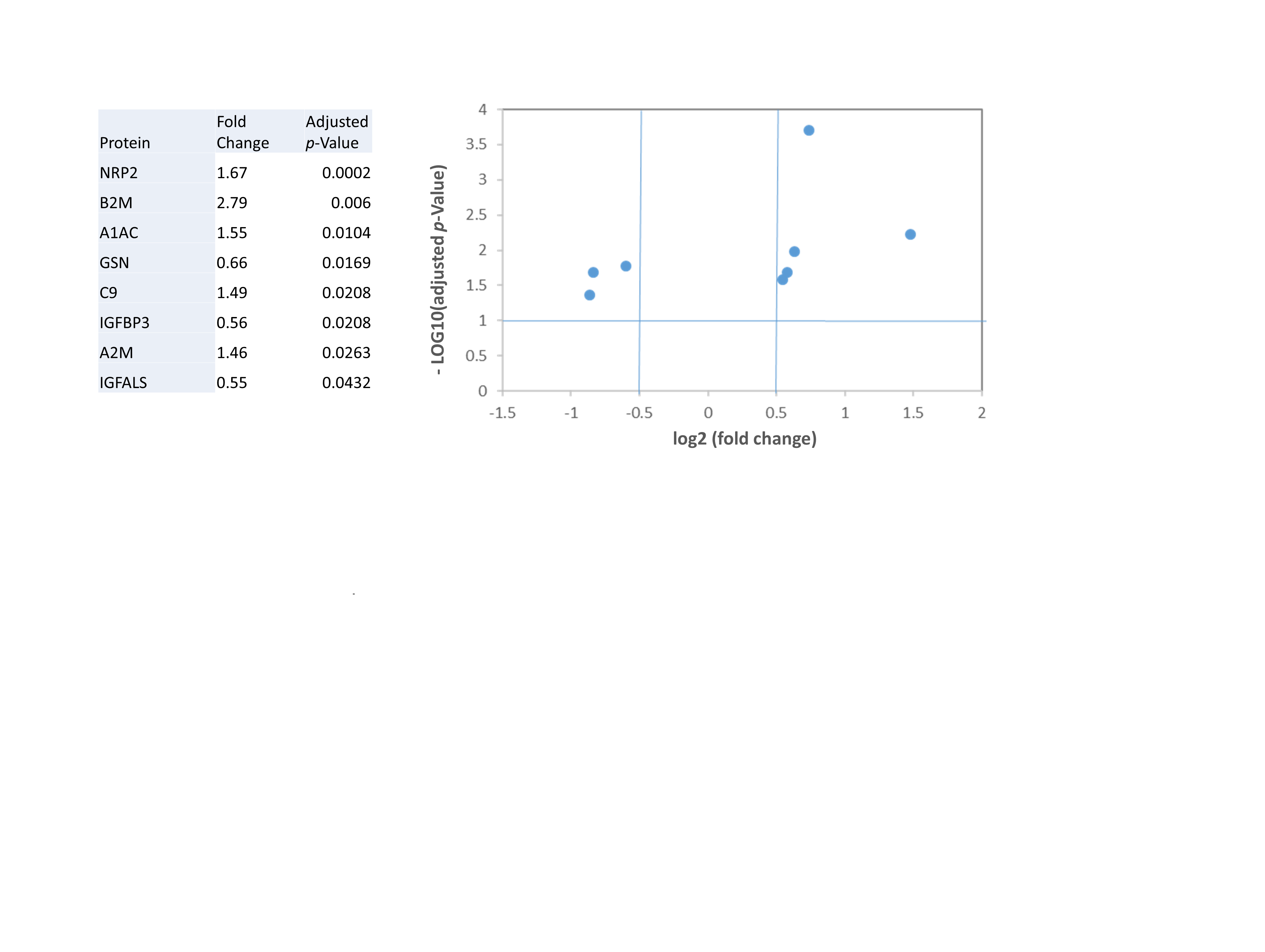

Supplement: Supplementary file 1 [file ijms-22-00838-s001.zip › Figure 2 4X.tif]

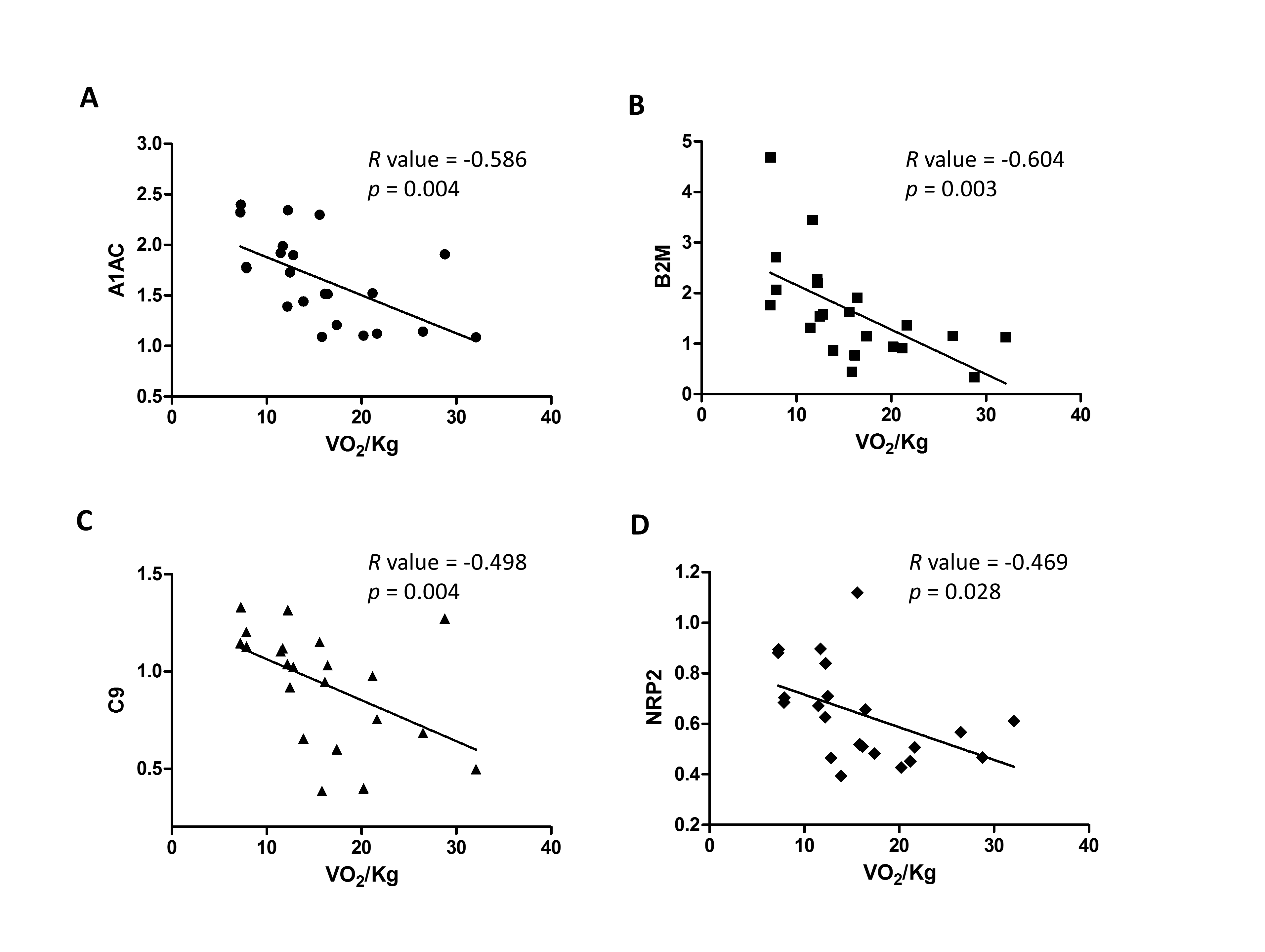

Supplement: Supplementary file 1 [file ijms-22-00838-s001.zip › Figure 3 4X.tif]
